# Reflections on Managing the Performance of Value-Based Healthcare: A Scoping Review

**DOI:** 10.34172/ijhpm.2023.7366

**Published:** 2023-05-31

**Authors:** Hilco J. van Elten, Steven W. Howard, Ivo De Loo, Frans Schaepkens

**Affiliations:** ^1^Nyenrode Business Universiteit, Breukelen, The Netherlands; ^2^Health Services Administration Department, School of Health Professions, University of Alabama at Birmingham, Birmingham, AL, USA

**Keywords:** Value-Based Healthcare, Performance Management, Healthcare Value Chain, Barriers to Adoption, Systematic Review

## Abstract

**Background:** Value-based healthcare (VBHC), which can be viewed as a strategy to organize and improve healthcare services, has far-reaching organizational and managerial consequences. It is common managerial practice to support the execution of a strategy by monitoring the ensuing activities. Such monitoring provides feedback and guidance on the execution of these activities to the management of an organization and helps to realize organizational strategies. Monitoring of activities is commonly done by performance management systems. Given the rising attention in the literature and in practice for VBHC, we ask to what extent VBHC is supported by performance management systems in practice, and how we can explain what we find to support further successful implementation of VBHC.

**Methods:** In our scoping review of financial and performance management at the organization or unit-level of healthcare organizations that apply value-based approaches, we identified 1267 unique papers in Embase, Medline, OVID, and Web of Science. After the (double-blinded) title and abstract screening, 398 full-text articles were assessed for further analysis.

**Results:** Our review reveals only eleven original papers discussing specifically the integration of VBHC and performance management systems. Almost all the featured applications in these papers focus on a specific project or medical specialty. Only one paper exemplifies how VBHC has been integrated with the performance management systems of a medical institution, and no paper provides a clear link with strategy execution. We ask why this is the case and propose several explanations by studying the extant performance management literature. We see these explanations as issues for further reflection for VBHC practitioners and researchers.

**Conclusion:** We conclude that one of the reasons for the absence of papers integrating VBHC and performance management systems is formed by the tensions that exist between striving for "the best care" or even for providing "all care that is viably possible" and pursuing greater (financial) efficiency. Implementing VBHC as an important organizational strategy and explicating this strategy in the performance management systems requires that these tensions need to be brought into the fore. When this is not done, we believe that VBHC adoptions that are fully integrated with performance management systems will remain limited in practice.

## Introduction

**Highlights**

- Value-based healthcare (VBHC) initiatives have become increasingly popular in healthcare organizations. The VBHC literature states that the performance of VBHC initiatives ought to be strictly monitored. This may happen by means of performance management systems. However, the monitoring of VBHC performance only seems to happen to a limited extent in practice and often within the confines of a single project or single organization, whereas the literature claims VBHC is most effective when it is implemented across the entire healthcare value chain.
- We suggest that existing tensions between doctors, patients, managers and administrators hinder a more integral, strategic adoption of VBHC in practice. These tensions may be caused by differences in interpretation of what ‘care’ entails. Is care purely or largely relational and procedural, or can it be expressed, even partially, in predefined monetary terms?
- We put forward the view that when the aforementioned tensions are insufficiently recognized and addressed, extensive VBHC adoptions that are fully integrated with performance management systems are not very likely, limiting the benefits that the adoption of VBHC may bring.

Value-Based Healthcare (VBHC) is often regarded an important innovation aimed at reforming healthcare practice and policy. It aims to improve healthcare outcomes whilst containing the costs of healthcare services. Despite the fact that many governments, including in the United States and in Europe, have increasingly focused on cost containment of healthcare services, healthcare expenses as a percentage of gross domestic product have been growing worldwide, often at rates surpassing the growth of countries’ gross domestic product.^1^ According to Porter and Teisberg,^2^ this is partially due to the way in which healthcare services are organized and managed. Among others, the measurement and management systems prevailing in healthcare organizations tend to suffer from inadequate incentives for healthcare staff, ineffective forms of competition between medical institutions, and incomplete information exchanges between stakeholders.

 In an attempt to mitigate these problems, Porter and Teisberg^3^ introduced VBHC, which they claimed would shift the focus and incentives prevailing in healthcare organizations from volume to value. Hence, they implied that adopting VBHC would lead to substantial strategic change in healthcare organizations, fundamentally affecting the way they operate. For instance, according to the VBHC-literature, value would have to be operationalized as health outcomes per Euro or Dollar spent. Outcomes would have to be defined in terms of what matters to patients — ie, what they care for and about. In a recent review of the literature, Zanotto et al find that measuring performance through generalized outcomes (eg, by looking at hospital mortality rates, infection rates, and medication errors) rather than by outcomes that matter to the patient, is common practice in medical institutions.^4^ This complicates the implementation of VBHC initiatives.

 In addition, VBHC initiatives should affect the entire healthcare value chain, and ought not to be confined to a single department, medical specialty or just a handful of organizations. The aforementioned focus on quality (ie, value) instead of quantity (ie, volume) has also been promoted by other recent initiatives in the healthcare sector, for example by the Triple Aim and accountable care organizations,^5,6^ but turns out to be difficult to effectuate. As noted, a focus on value instead of volume has far-reaching strategic and organizational consequences. Adopting a VBHC-based strategy changes not only the internal organization of healthcare processes, but also the contractual relations with those who pay for healthcare services. Obviously, the internal organization of healthcare processes has to be aligned with the development of contractual relations. When a medical facility decides to adopt VBHC, healthcare insurance companies may find this an ideal opportunity to introduce incentives in their payment schemes that promote the shift from a pure volume-based scheme to a value-oriented care delivery structure.^7^ Such a shift in payment scheme could strongly influence the revenues of healthcare providers,^8^ but also the internal organization of healthcare processes. Zanotto et al^4^ therefore call for a culture in managing of medical institutions, which we associate here with the development and implementation of performance management systems, as will be explained below.

 Despite the far-reaching (cross) organizational consequences of adopting VBHC, very little is known about the practical implications of adopting VBHC, for example in hospitals or psychiatric wards.^9^ Much of the associated literature has been written by consultants who tend to focus on success stories. A potentially insightful theoretical viewpoint on VBHC adoptions, how these may be effectuated, and the challenges they involve is provided by the performance management literature. Among others, this literature suggests that well-designed performance management systems are needed to monitor organizational performance and to steer and direct the actions of managers and other organizational members to safeguard the strategic direction of the organization.^10,11^ Otley^12^ defines performance management as a set of activities aimed at ensuring the alignment of organizational objectives, its strategies, activities, performance targets across various levels of the organization, performance measurement, and reward systems.^13^ In this study, performance management involves defining, measuring and evaluating healthcare outcomes (eg, Patient Reported Outcome Measures, Patient Reported Experience Measures, clinical outcomes, vaccination rates, screening rates — ie, both outcome and process measures) and related expenses, as well as utilizing this information in a management context (eg, in dashboards and benchmarks, and for making strategic and operational decisions). van Veghel et al^14^ argue that by managing performance, those working in healthcare organizations receive information and incentives that give important insights into, and will subsequently affect, their contribution to organizational strategy, which in a VBHC setting is assumed to focus on improving patient value.

 Our research question is formalized as: to what extent is VBHC supported by performance management systems in current practice, and how can we explain our findings? To substantiate our explanations, we draw some general insights from the extant performance management literature and relate this to the adoption of VBHC. We then consider various practical managerial implementations for VBHC adoptions. This implies that in our scoping review of the literature, in which we aim to summarize what is known empirically about this question, we address the implementation of VBHC in medical facilities from a performance management perspective.

 If the insights stemming from our scoping review are indeed supporting the claims made by advocates of VBHC about increased value for patients, we will have a better idea of how to integrate VBHC and performance management systems so that the benefits of VBHC may (more easily) be brought into the fore in the future. If the claims are unsupported, we will try to argue (ie, find potential explanations) why this is the case. A scoping review, we argue, is a promising way to see what has been reported about VHBC and performance management in practice, as it allows us to analyze and summarize the extant literature by means of a systematic review process.

## Methods

 Our research consists of two parts; the scoping review resulting in the selection of eleven papers — which is described in the current section of the paper — and a literature-based interpretation of these findings (using the performance management literature).

 Our scoping review research methodology is based on the Preferred Reporting Items for Systematic Reviews and Meta-analysis Protocols for scoping reviews (PRISMA-ScR). Scoping reviews are appropriate for identifying key characteristics and factors related to a concept.^15^ They are often used for mapping out the evidence base pertaining to a particular research question or topical area.^16^

 As our research addresses the research topic of performance management of VBHC, we conducted a systematic search across three databases (Embase, Medline OVID, and Web of Science) to examine the empirical evidence base in the extant literature. Our systematic search consisted of four phases. We discuss these phases and the related research findings (ie, a description of the general characteristics of the relevant papers that were distinguished) in the next section. This is followed by an in-depth, content-related description of the research findings. Here, we look specifically at what the papers have to say about VBHC and performance management. Thereafter, a literature-based interpretation of these findings follows. As we found very few papers discussing performance management issues in VBHC-settings (as will be set out below), we ask why this may have been the case. In answering this question, we also take our cues from the performance management literature, looking at potentially relevant discussion points that we believe may apply to VBHC initiatives. Finally, we will present our reflections on the research.

## Results: Findings of the Scoping Review

 First, a specific search strategy (See Supplementary file 1) was developed, tailored to the three databases mentioned in the previous section. The strategy was applied at the end of January 2021 and updated at the beginning of November 2021. This search yielded 1267 papers (excluding 376 duplicates), as displayed in the PRIMSA flowchart in Supplementary file 2. Second, titles and abstracts were blindly screened by a team of authors and five research assistants. The research assistants were carefully instructed by one of the authors, and the screening process was subject to multiple rounds of calibration between the primary researchers (ie, the authors of this paper) and the research assistants. All titles and abstracts were randomly assigned to two of the team members, and the assessment of the titles and abstracts was administered by the first author. The interrater reliability was considered moderate but reasonable (Cohen’s kappa = 0.6366). The screening by the authors and research assistants was conducted blindly and independently. 869 papers were excluded during this phase, based on the assessment of titles and abstracts. Third, the remaining 398 full-text papers were screened in-depth. Similar to the title and abstract screenings, full texts were assigned randomly to two of the team members. The relevance of each study was assessed by using the inclusion criteria presented in Table 1. Any discrepancies between research assistants were resolved by consensus. Studies that did not meet the preset criteria were excluded; the reasons for their exclusion have been summarized in Table 1. Since exclusion criteria are not mutually exclusive, we report both reviewers’ first observed reason for each excluded full text (See Supplementary file 2). The top-three reasons for excluding papers during this phase were: (1) the paper’s focus on value-based procurement, purchasing and/or reimbursement, rather than VBHC (n = 199). As we are primarily interested in the performance management of VBHC initiatives, we focus on internal organizational aspects of managing such initiatives. Value-based procurement, purchasing and/or reimbursement research — although conceptually related — primarily look at relations between an organization and relevant outside parties, such as insurers and governments. Therefore, procurement, purchasing and/or reimbursement are outside the scope of our review. Other reasons for exclusion were: (2) the paper’s lack of original research, being either conceptual, theoretical or editorial (n = 121), and (3) the absence of a full-text version of a paper (n = 116). The full-text assessment led to 387 excluded and 11 included papers.

**Table 1:** Inclusion and Exclusion Criteria

**Inclusion**	**Exclusion**
VBHC	Value-based procurement, value-based reimbursement, value-based purchasing
Healthcare organizations (eg, hospitals, nursing homes)	Organizations that do not provide care or cure themselves, eg, pharmacies and healthcare insurance companies
Financial and performance management at the organization or unit-level (eg, not individual doctors or countries)	Economic evaluations; patient level performance measures (eg, PROMs) not related to organizational performance
Full-text available	Full-text unavailable
Empirical, original research	Reviews, simulations, research protocols, editorials
English or Dutch	Other languages

Abbreviations: VBHC, value-based healthcare; PROMs, patient reported outcome measures.

 Fourth, the eleven remaining papers were read and analyzed by each of the primary researchers (ie, three of the four authors of this paper), for quality assessment and data extraction. The data extraction was aimed to qualitatively summarize the included papers in terms of their aim and scope, year of publication and geographical area, and the papers’ main findings. We supported the data extraction with a spreadsheet document, completed independently by three out of four of the primary researchers. The summarized data extraction is presented in Table 2. A separate data extraction sheet (not tabulated) supported our qualitative assessment of the included papers. In addition, the sheet helped us to generate an overview of the available empirical evidence on the performance management of VBHC.

 Next, we provide some general observations on the included papers. These observations will be elaborated upon thereafter. We will subsequently address the associated empirical evidence base in the performance management literature, identifying issues or points of discussion that we feel need to be tackled to foster VBHC initiatives in healthcare organizations and across the healthcare value chain.

**Table 2:** Data Extraction

**Authors**	**Year**	**Country**	**Research Method**	**Department/Subject/Disease**	**Aim According to Paper**	**Main Results/Suggestions**
Bonde et al^23^	2018	Europe (Denmark)	Qualitative case study	General diagnostic centre; neurology; otolaryngology; dental surgery; orthopaedic surgery; endocrinology; ophthalmology; and emergency medicine	To analyze a VBHC inspired experiment called the 'New Governance in a Patient perspective' implemented in 9 departments of a hospital.	Bottom-up approach starting from notions of care and value, and reliance on locally developed performance measures using ‘dialogical accountability.’
Côte-Boileau et al^24^	2021	Canada	Qualitative study – Multisite organizational ethnographic case studies and narrative process analysis	Integrated (academic) health and social services centres	To explore how do organizational actors appropriate control rooms as managerial tools to influence value-based performance in health systems.	There is no ‘one-size-fits-all’ solution for VBHC implementations, but consideration for implementing, testing, adapting the right mechanisms to support so-called control rooms is a first, important step.
Feitz et al^25^	2021	Europe (The Netherlands)	Quantitative study – longitudinal analysis of patient, doctor, and therapist data	Integrated private hand surgery clinic offering public service	To use outcome data to improve care by returning outcome data to patients (for shared decision-making, patient selection by baseline thresholds, individual prediction of treatment results, and outcome progression over time for an individual patient), to surgeons and therapists (for feedback loops facilitating ongoing learning, and for extreme value detection to intervene when needed).	Feedback loops can be used to improve shared decision-making, to monitor or predict treatment progression over time, for extreme value detection, and surgeon evaluation.
Nycz et al^26^	2020	USA (Wisconsin)	Qualitative-Retrospective data analysis	Dental care	To establish the impact of implementing data-driven performance metric-tracking across a 10-dental centre infrastructure for relative impact on achieving value-based care delivery.	Implementing a quality metric dashboard and a 1:1 dentist-to-hygienist ratio supports the realization of value-based dental care delivery.
Parra et al^17^	2017	Europe (Spain)	Quantitative case analysis	Renal Unit, Internal Medicine	To develop a methodology for assessing the value of healthcare delivered by haemodialysis centres according to a comprehensive, feasible set of outcomes.	The developed value methodology encourages clinicians to produce circumstances that are most relevant to patients. Value perceived by the patient does not necessarily always relate to evidence-based medicine, but also includes patient satisfaction with a medical centre.
Reilly et al^18^	2020	USA (New Hampshire)	Quantitative-Retrospective case analysis	Department of Orthopaedics	To develop a value dashboard with benchmarking that displays value and gives insight into potential value-adding activities of total hip and knee arthroplasty (THA and TKA).	The proposed dashboard can be used to evaluate the value of TKAs and THAs. The dashboard combines patient-outcome metrics, both clinical and patient-reported, and direct costs of surgical procedures. Regional and national benchmarking could provide a more accurate representation of value, as opposed to using institutional averages.
Robinson et al^19^	2018	USA (Tennessee)	Prospective observational study	Department of Pediatric Surgery	To evaluate the effect of providing individual surgeons with data feedback on outcomes and costs (value). To evaluate the impact of cost and outcome feedback to surgeons on value of care in an environment reluctant to adopt a standardized surgeon preference card.	Approaching value measurement with a surgeon-specific, rather than a group-wide standardization approach, providing real-time cost data to surgeons, and changes their behavior significantly even without additional incentives.
Van den Berg et al^20^	2020	Europe (The Netherlands)	Quantitative study – Retrospective cohort study	Obstetric cooperations of two recently merged hospitals, and a number of affiliated but independent midwifery practices.	To demonstrate a practical approach to VBHC for obstetrics and demonstrate what is necessary to learn through benchmarking.	Defining, measuring, and comparing relevant outcomes enable care providers to identify improvements. Continuous monitoring of clinical outcomes and expanding the set of outcomes that is readily available to medical specialists are key in the process towards value-based care provision.
Van der Nat et al^27^	2021	Europe (The Netherlands)	Quantitative-survey research	Heart centres in Dutch hospitals	To give an up-to-date assessment of outcome-based quality improvement in 2020 at a national level in Dutch heart care.	Health outcomes have become a relevant element in quality improvement and organization of Dutch heart centres. Heart centres are more able to use the acquired insights based on these measurements to initiate improvement projects, showing potential for heart centres to share best practices in the implementation of VBHC.
Winegar et al^21^	2019	USA (Texas)	Quantitative-Retrospective case study	Surgery and Perioperative care, Orthopaedics	To examine to what extent an unblinded orthopaedic surgeon-specific value scorecard improves patient outcomes and lowers hospital costs.	The scorecard resulted in reduced costs, and reduced postoperative length of stay, without compromising clinical outcomes. Sharing unblinded clinical and financial outcomes with surgeons may promote a culture of shared accountability and may empower surgeons to improve value-based decision-making in care delivery.
Zipfel et al^22^	2020	Europe (The Netherlands)	Single case study	Department of Cardiology	To develop a toolbox for selecting improvement interventions that positively influence health outcomes in the right direction. The secondary aim is to apply this toolbox to aortic valve disease.	The toolbox turns out to offer a practical guide on how to identify and select improvement interventions based on VBHC.

Abbreviations: VBHC, value-based healthcare; THA, total hip arthroplasty; TKA, total knee arthroplasty.

 Our first observation concerns the limited number of papers in the topical area at hand. We indicated that performance management is considered to be of vital importance in the pursuit of organizational strategy. Since the adoption of VBHC is supposed to have far-reaching internal and external consequences for healthcare organizations and the link with performance management has been implied by VBHC protagonists, we would have expected this theme to be heavily featured in the extant literature.^11^ Despite the massive impact that VBHC has on how healthcare organizations are run,^2,3^ we only uncovered eleven papers^17-27^ that addressed the performance management of VBHC. None of these specifically discussed the strategic implications of adopting VBHC, suggesting that healthcare organizations’ adoption of VBHC is less all-encompassing than what its protagonists claim is warranted. This less than all-encompassing adoption is supported by the fact that all but one of the papers are limited in scope to a project, department, specialty or time period.

 In addition, we observe that empirical research on the performance management of VBHC initiatives stems predominantly from Europe^17,20,22,23,25,27^ and the United States.^18,19,21,26^ The overrepresentation of Europe and North America is not uncommon to the VBHC literature, although most review articles on VBHC also contain papers from other parts of the world, see for example Keel et al^28^ and Reitblat et al.^29^

 Although the search strategy was not confined to hospitals, all the included papers study VBHC in hospital settings. None of the papers looked specifically at other healthcare providers, and only intermittently reference was made to other medical facilities.

 Furthermore, we find that all eleven included papers are relatively recent (>2015), despite the fact that our search was not limited to a specific time period. We also find an increase in the number of papers (of over 50%) in 2020 and 2021. The findings suggest that links between the performance management and VBHC literature are relatively new and may be developing, even though the VBHC literature itself dates from the beginning of this century, and such developments might have been expected to have popped up in the literature earlier.

 Finally, all included papers present ‘successful’ VBHC projects and implementations. For publication reasons, it may be that ‘successful’ VBHC initiatives are preferred over ‘not that successful’ ones. However, one may learn more from ‘not that successful’ cases in which things did not turn out as planned than from cases of projects that were highly successful. To date, VBHC initiatives have also largely been entrepreneurial/experimental, with many practitioners emphasizing the value of learning from their VBHC experiments. The literature in the field of entrepreneurship has shown evidence of publication bias in favor of successful endeavors.^30-32^ We put forward the view that there should be more room for publication of studies wherein the value was more learning-related than direct health outcome or economic benefit.

## Results: The Relation Between Performance Management and VBHC

 In this section, we describe what the included papers had to say about the use of performance management in VBHC settings. Thereafter, we interpret these findings using the extant performance management literature, setting out potential points of discussion that we believe may have hampered the integration of performance management systems in concrete VBHC applications.

 Two general approaches of how to implement VBHC, specifically in hospitals, can be found in the literature: a top-down and a bottom-up approach. The top-down approach is based on Porter and Teisberg,^2^ Porter and Teisberg,^3^ who assert that given a patient-derived definition of ‘value,’ detailed VBHC initiatives (including performance management systems) can be successfully rolled out across the healthcare value chain via a clear sequence of steps — provided that sufficient care is taken during the implementation process. If such care cannot be guaranteed, VBHC initiatives can easily falter. The second approach, which sharply contrasts the approach put forward by Porter and Teisberg,^2^ Porter and Teisberg,^3^ is discussed by Bonde et al.^23^ These authors sketch a bottom-up approach, starting from notions of care and value stemming from and sustained by patients, which are picked up and supported by medical specialists, who actively engage in building performance management systems, tools and techniques that safeguard these notions of value and care, and help to provide valuable management information. Côté-Boileau et al^24^ show, in their ethnographic research, that the two approaches can be mixed: some planning is necessary for VBHC implementations to be set in motion, but many decisions in the VBHC cases they studied were made as the implementation of VBHC developed. Naessens et al^7^ assert that the role of insurance companies and healthcare policy-makers (and external stakeholders more generally) when implementing VBHC initiatives may be larger than is commonly surmised or appreciated by VBHC supporters, and that these stakeholders may exert considerable influence on how VBHC implementations develop. Similarly, the regulatory environment and type of national health system may impact VBHC design and implementation. Earlier research has acknowledged that VBHC implementations are highly complex since they straddle the entire healthcare value chain, and are contingent on a variety of structural, technical and cognitive factors that cannot all be controlled or foreseen. Therefore, there exists no ‘one-size-fits-all’ approach suiting VBHC implementations.^24^

 The included papers display a variety of performance management issues and concepts. Most papers report on the reliability of performance measures of clinical outcomes,^17-22,25,26^ patient reported outcomes,^17,18,20,21,23^ and/or costs of hospital departments or of particular healthcare processes (eg, purchasing).^17-19,21^ The reported performance measures all seem to have provided valuable information for managerial decision-making, although this is not discussed at length in any of the papers. One of the papers^24^ provides an extended narrative, based on ethnographic research, on how a set of Canadian hospitals developed, implemented and tested the performance instruments that they put into practice in a VBHC pilot supported by the Canadian government. The authors give special attention to so-called control rooms, ie, spaces where stakeholders regularly met to discuss VBHC-related issues and decided how to communicate their efforts and decisions to others involved in the VBHC-pilot. A totally different paper in our sample stems from van der Nat et al.^27^ This is a national (survey) study on VBHC implementation in heart centres in the Netherlands. Even though Dutch heart centres have been said to be frontrunners in adopting VBHC, these adoptions are still relatively limited and differ substantially between the centres studied. Consequently, the performance management concepts the centres apply and the issues they run into tend to differ, which is lamented by the authors as the standardization of VBHC efforts is delayed. In the included papers, various tools, techniques and technologies are mentioned to manage healthcare performance, illustrating how VBHC may be effectuated in concrete situations. Feitz et al^25^ provide a longitudinal study, discussing the development in tools and techniques they witnessed over a 10-year period in hand surgery as they tried to organize their work using VBHC principles. Robinson et al,^19^ Winegar et al,^21^ and Reilly et al^18^ rely on ‘value scorecards’ representing the performance of individual surgeons. Another frequently used performance management tool is benchmarking.^18,20,22,26^ Another paper does not discuss benchmarking in a detailed way, but does acknowledge the benefits that may be derived from its application.^17^

 Several papers address key performance indicators (KPIs) of VBHC that are contained in performance management systems and the way in which these have been determined. KPIs are often chosen to ease analyses across hospitals. Interestingly, according to some papers,^17,18,20,25,26^ medical specialists and their managers ought to be allowed to choose (the majority of) appropriate KPIs, even though VBHC focuses on what matters to patients. In one of the papers, Reilly et al,^18^ it is asserted that through their interactions with patients, specialists develop sufficient practical wisdom to know what matters to their patients, leading to easier identification of relevant KPIs. Hence, they should be involved in choosing and setting KPIs and KPI standards. Occasionally, KPIs are based on the KPIs present in generally available medical reports,^33^ facilitating comparison between hospitals.

## Interpretation of the Findings

 When comparing the insights about performance management mentioned in the previous section with what one of the most influential researchers in performance management, Otley^12^ has termed ‘performance management,’ we see that target setting and performance measurement are emphasized in the papers included in our analysis. However, among the papers in our scoping review, there is little discussion about healthcare institutions’ key objectives, their strategies, and reward systems, which Otley claims are all important elements of performance management. According to Otley,^12^ these elements always need to be aligned with the setting of targets and the measurement of performance across various levels of the organization for performance management systems to function properly. This alignment would therefore also be important for VBHC initiatives to function well. The focus in the extant literature is firmly on the development of performance measures and the results VBHC applications have led to. One may wonder why the other topics that Otley^12^ mentions remain undiscussed in the extant literature. We offer two potential explanations below.

 First, VBHC is to be implemented at the level of the entire healthcare value chain rather than within a single or just a few organizations to reap the benefits of its implementation. The fact that all of the included papers were confined to one type of institution in the chain (ie, hospitals, and projects within hospitals) suggests that cross-organizational cooperation, which is one of the characteristics of VBHC, may still be in its infancy, at least as far as performance management systems are concerned. Alternatively, given the focus on ‘successful’ case studies in the literature, it may be that other VBHC implementations across the healthcare value chain have proven notoriously difficult and/or unrealistic to succeed, and have consequently not been reported. The focus in the eleven papers analyzed in this research lies largely on the technical aspects associated with implementing VBHC, ie, the choice of performance instruments, KPIs and the results stemming from VBHC implementations. VBHC has wide-ranging organizational effects and moves well beyond mere quantification, although quantification is heavily featured in the papers at hand. Apart from two studies,^7,24^ relatively few details are provided about how VBHC implementations went and developed, which adaptations were required over time, who was involved therein, and what the views of patients were who were confronted with changes in healthcare associated with implementing VBHC. Perhaps the presence of many quantitative studies in our sample can (partially) explain this outcome: these studies rarely adopt an implementation process perspective. This is the second reason why we believe that some topics that Otley^12^ finds important when discussing performance management may not have been addressed very much in the VBHC literature to date.

 Hopwood,^34^ who is another influential researcher in performance management, claims that our everyday lives have become increasingly performance managed: among others, we have gotten used to talking about each other and what we do in terms of assets, liabilities, debts, balances, and/or value. In that sense, it is understandable that healthcare is progressively being presented and assessed in such terms as well. At the same time, as our scoping review suggests, there may be limited, full-blown applications of VBHC in practice, despite the concept having been around for almost two decades. What could explain this paradox, if we assume that the VBHC literature is not overly optimistic in its claims? We suggest that there may be tensions between care, caring, and the quantification and standardization of care, which is part and parcel of VBHC,^35^ leading to the limited number of VBHC adoptions we currently see discussed in the literature. We believe these tensions may also offer another explanation for the lack of integration between VBHC and performance management found in practice, apart from the integration being at its infancy. Notions of care and caring imply that patients ought to be approached and handled by, for instance, medical specialists, in a way that supports their recovery or alleviates their pain and/or distress; whilst specialists have to do their best to make this happen. How this is put into practice exactly is to be determined in continuous interaction and debate between the individuals involved,^35^ who have to apply their professional skills and knowledge when engaging in these interactions. To what extent does this really happen in practice? Naessens et al^7^ argue that performance measures in VBHC tend to be based on easy to obtain data that do not necessarily reflect the complexities of everyday healthcare practice, which may intensify the aforementioned tensions and contribute to the lack of integration between VBHC and performance management (See also: Otley^12^). The papers we examined, with one exception,^23^ do not address the difficulties involved in developing KPIs that do sufficient justice to the complexity of care.^27^ On the one hand, focusing on cost containment in a sector with costs that frequently spiral out of control is understandable.^1^ But, as noted, such an approach is bedeviled with tensions and difficult decisions: among others, about whether particular treatments ought to be provided to a particular patient at a particular moment in time, which treatment(s) ought to be provided anyway in case of a certain condition, how frequently, for how long, using which standards of assessment, etcetera. Issues such as these are not easily resolved; stakeholders need to be willing to carry the consequences of the decisions they take involving these issues. There is a danger that whatever is contained in or suggested by a performance management system is deemed to be the ‘best’ way to go in a certain situation and that decisions are made accordingly, but this ignores the fact that care and caring is principally a human activity and not primarily a numerical exercise.^35^ This may be another reason why medical facilities are reluctant to integrate VBHC and performance management, or to introduce specific performance management systems tailored to VBHC too quickly or extensively. However, we acknowledge that what is clinically needed to care for a patient has to be set against the financial costs, the allocation of scarce resources, and the demands by insurers and government for efficiencies (See also Zuurbier^36^) when VBHC initiatives are effectuated. We would like to see more papers that focus on the associated decisions in the performance management and VBHC literature.

## Concluding Remarks

 Valuing something involves quantification, whilst (health)care in itself does not.^35^ This makes VBHC an intriguing and challenging concept that may be difficult to implement. It is intriguing because VBHC allows the management of healthcare from a distance (by hospital managers), using specific measurements, techniques and tools that may be compared between medical specialists, hospital departments, and medical institutions more generally. VBHC is challenging because quantification cannot happen without the dilemmas and difficult decisions that we have previously discussed. The implementation of VBHC may be wrought with issues since it needs to cover the entire value chain. In addition, it should be kept in mind that performance management has performative effects that warrant discussion and in-depth analysis as well.^37^ There is no (single) ‘right’ or ‘wrong’ way to implement VBHC, and the complex nature of VBHC implementations means that they must be handled with care and cannot be completely prescribed.^24^ A clear notion of ‘maximizing patient value’ needs to be taken into account and periodically checked, and performance management systems have to be built that safeguard the progress of VBHC initiatives in line with Otley,^12^ if VBHC implementations are to be developed in line with the performance management literature. However, when 1-on-1 contacts with patients are deemphasized (on the assumption that one can ‘know’ from a distance what adequate care looks like), VBHC may move in a direction that does more harm than good to one’s healthcare efforts, no matter how impressive the associated financial results may seem and/or how satisfied hospital management may look. After all, when a top-down approach to VBHC is followed, discussions about the complexity of healthcare will not automatically come into the fore as the definition of care that is to be upheld is defined from the outset. When following a bottom-up approach, however, it is inevitable to frequently have such discussions, as prevailing notions of care are to be based on the actual, current desires and experiences of patients, which may change from patient to patient, over time, from situation to situation, and possibly also from specialist to specialist. In order to resolve this issue, perhaps, it could be attempted to straddle the ‘top-down’ and ‘bottom-up’ approach of implementing VBHC.

 Almost all countries struggle with increasing healthcare expenses. This struggle is intensified by demographic trends such as the aging of populations, and the subsequent, increasing demands for healthcare.^1^ Hence, concepts such as VBHC are appealing to governments, healthcare policy-makers, insurance companies and medical institutions. It is generally acknowledged that performance management is beneficial to reap the benefits of (for organizations) impactful initiatives such as VBHC.^38^ However, based on the included papers, there have only been small-scale experiments with the strategic implementation of VBHC, and the installment of appropriate performance management systems. We also found no applications that considered the entire healthcare value chain. Existing applications avoid many of the dilemmas mentioned above and even though they have all been called a ‘success,’ they have only had a positive impact in a narrowly defined sense (within a single department or centre, when purchasing specific medical tools, etcetera). Such initiatives, therefore, are not extensive enough or sufficiently encompassing to break the trend of ever-increasing healthcare expenses.

 Deciding to integrate VBHC and performance management systems will create tensions, since medical specialists and patients will generally aim for the “best possible care” and for “all care that is possible,” whereas administrators and managers chiefly tend to care about attaining budget targets and the general distribution of care across medical disciplines and patients. Côté-Boileau et al^24^ suggest that setting up VBHC ‘control rooms’ may be an effective way to mitigate these tensions, although the authors acknowledge that they mainly studied those who were engaged in the control rooms, and not so much those who were affected by the decisions taken in these rooms. Choosing not to fully engage in cost containment efforts because of the potentially difficult aspects of embedding VBHC in performance management systems implies that one is willing to just muddle through, hoping for practitioners to put their efforts into designing better experiments and other small improvement projects, which, we believe, will never be able to reap the potentially substantial and lasting benefits of VBHC that are proclaimed in the literature, promising more value added for patients.

## Acknowledgements

 The authors thank our graduate students Victorien Luppes, Mathijs Carvalho Mota, Marije Strikwerda, Lequisha Suisse, and Koen Wijsman for their assistance in the screening process.

## Ethical issues

 This study was exempt from IRB approval. Guidelines deem it outside the scope of the Netherland’s Medical Research Involving Human Subjects Act because it did not concern “medical/scientific research” about illness and health, nor did the content or methods cause “an infringement of the physical and/or psychological integrity” of the participants. Therefore, according to Dutch law, no formal ethical approval was needed.

## Competing interests

 Authors declare that they have no competing interests.

## Supplementary files

Supplementary file 1. Search String.

Supplementary file 2. PRISMA Flowchart.
