# Supplementary material for: Reflections on Managing the Performance of Value-Based Healthcare: A Scoping Review
Source: Int J Health Policy Manag. 2023 May 31;12:7366. doi: 10.34172/ijhpm.2023.7366 (PMC10461846; doi:10.34172/ijhpm.2023.7366)
Supplement: Supplementary file 1 — Search String. [file ijhpm-12-7366-s001.pdf]

Article title: Reflections on Managing the Performance of Value-Based Healthcare: A Scoping Review

Journal name: International Journal of Health Policy and Management (IJHPM)

Authors' information: Hilco J. van Elten[1], Steven W. Howard[2]*, Ivo De Loo[1], Frans Schaepkens[1]

[1]Nyenrode Business Universiteit, Breukelen, The Netherlands.

[2]Health Services Administration Department, School of Health Professions, University of Alabama at Birmingham, Birmingham, AL, USA

(*Corresponding author: SHoward3@UAB.edu)

Supplementary file 1. Search String

Embase (dd 04-11-2021): 402

('value based':ab,ti OR valuebased:ab,ti OR vbhc:ab,ti OR 'vb hc':ab,ti) AND (kpi:ab,ti OR 'key performance indicator':ab,ti OR ((performance NEXT/2 (measure* OR manage* OR indicat* OR evaluat*)):ab,ti) OR ((management NEXT/2 (account* OR control*)):ab,ti) OR (target NEXT/2 setting) OR benchmark* OR dashboar*)

Medline OVID (dd 04-11-2021): 446

(value based or valuebased or vbhc or vb hc).mp. and (kpi or key performance indicator or performance measure* or performance manage* or performance indicat* or performance evaluat* or management account* or management control* or target setting or benchmark* or dashboar*).ab,ti. [mp=title, abstract, original title, name of substance word, subject heading word, floating sub-heading word, keyword heading word, organism supplementary concept word, protocol supplementary concept word, rare disease supplementary concept word, unique identifier, synonyms]

Web of science (dd 04-11-2021): 795

(TS=((value-based OR valuebased) AND ((kpi OR key performance indicator OR (performance NEAR/2 measure*) OR (performance NEAR/2 manage*) OR (performance NEAR/2 indicat*) OR (performance NEAR/2 evaluat*) OR (management NEAR/2 account*) OR (management NEAR/2 control*) OR (target NEAR/2 setting) OR benchmark* OR dashboar*))) AND DT=(article) AND LA=(dutch OR english))
