# Supplementary material for: Reflections on Managing the Performance of Value-Based Healthcare: A Scoping Review
Source: Int J Health Policy Manag. 2023 May 31;12:7366. doi: 10.34172/ijhpm.2023.7366 (PMC10461846; doi:10.34172/ijhpm.2023.7366)
Supplement: Supplementary file 2 — PRISMA Flowchart. [file ijhpm-12-7366-s002.pdf]

Article title: Reflections on Managing the Performance of Value-Based Healthcare: A Scoping Review

Journal name: International Journal of Health Policy and Management (IJHPM)

Authors' information: Hilco J. van Elten[1], Steven W. Howard[2]*, Ivo De Loo[1], Frans Schaepkens[1]

[1]Nyenrode Business Universiteit, Breukelen, The Netherlands.

[2]Health Services Administration Department, School of Health Professions, University of Alabama at Birmingham, Birmingham, AL, USA

(*Corresponding author: SHoward3@UAB.edu)

Supplementary file 2. PRISMA Flowchart

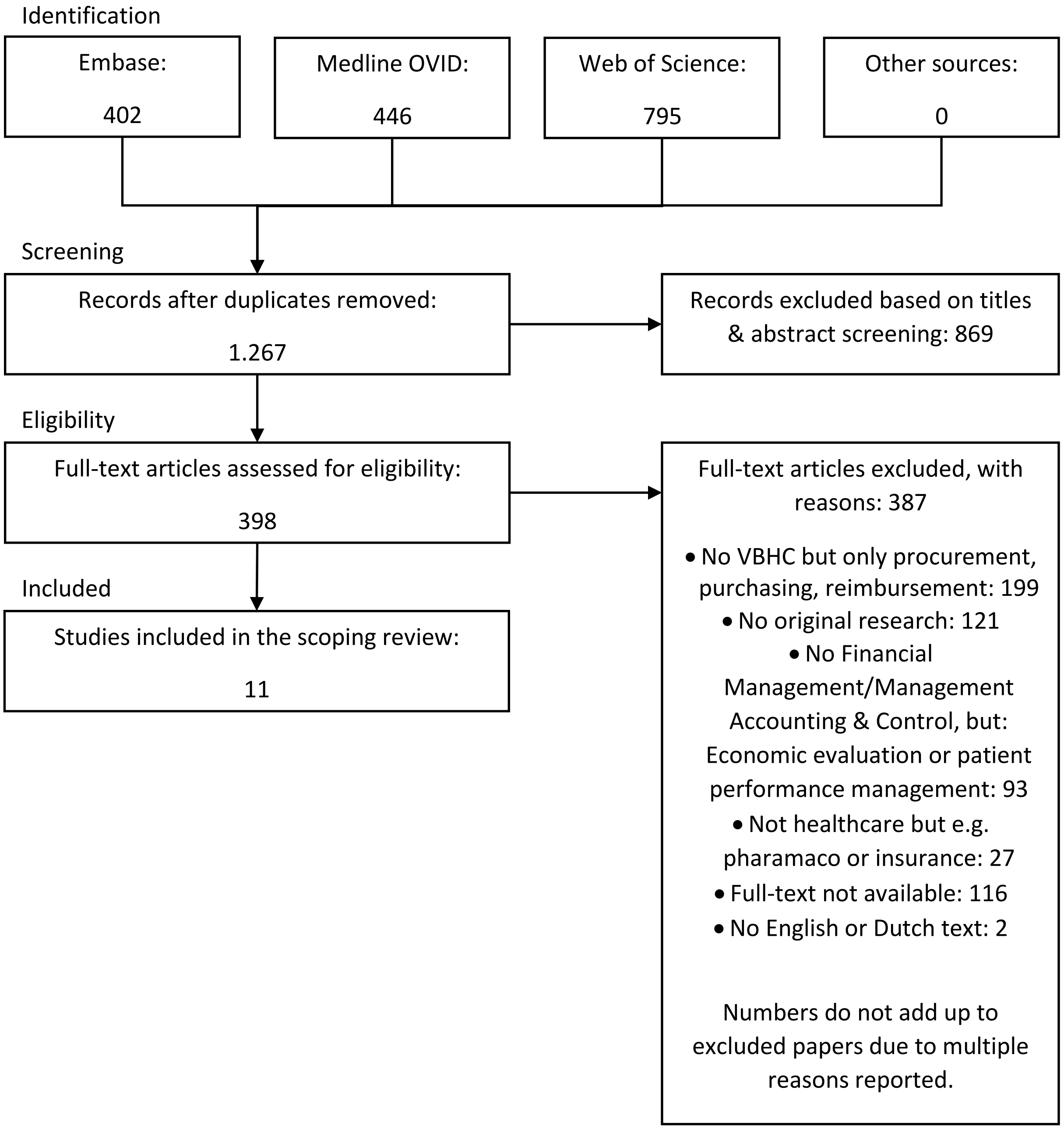
Identification
Embase: 402
Medline OVID: 446
Web of Science: 795
Other sources: 0
Screening
Records after duplicates removed: 1.267
Records excluded based on titles & abstract screening: 869
Eligibility
Full-text articles assessed for eligibility: 398
Full-text articles excluded, with reasons: 387
• No VBHC but only procurement, purchasing, reimbursement: 199
• No original research: 121
• No Financial Management/Management Accounting & Control, but: Economic evaluation or patient performance management: 93
• Not healthcare but e.g. pharamaco or insurance: 27
• Full-text not available: 116
• No English or Dutch text: 2
Numbers do not add up to excluded papers due to multiple reasons reported.
Included
Studies included in the scoping review: 11
